# Microglial Phagocytosis and Its Regulation: A Therapeutic Target in Parkinson’s Disease?

**DOI:** 10.3389/fnmol.2018.00144

**Published:** 2018-04-27

**Authors:** Elzbieta Janda, Laura Boi, Anna R. Carta

**Affiliations:** ^1^Department of Health Sciences, Magna Graecia University, Catanzaro, Italy; ^2^Department of Biomedical Sciences, University of Cagliari, Cagliari, Italy

**Keywords:** microglia, phagocytosis, cytokines, alpha-synuclein, parkinson

## Abstract

The role of phagocytosis in the neuroprotective function of microglia has been appreciated for a long time, but only more recently a dysregulation of this process has been recognized in Parkinson’s disease (PD). Indeed, microglia play several critical roles in central nervous system (CNS), such as clearance of dying neurons and pathogens as well as immunomodulation, and to fulfill these complex tasks they engage distinct phenotypes. Regulation of phenotypic plasticity and phagocytosis in microglia can be impaired by defects in molecular machinery regulating critical homeostatic mechanisms, including autophagy. Here, we briefly summarize current knowledge on molecular mechanisms of microglia phagocytosis, and the neuro-pathological role of microglia in PD. Then we focus more in detail on the possible functional role of microglial phagocytosis in the pathogenesis and progression of PD. Evidence in support of either a beneficial or deleterious role of phagocytosis in dopaminergic degeneration is reported. Altered expression of target-recognizing receptors and lysosomal receptor CD68, as well as the emerging determinant role of α-synuclein (α-SYN) in phagocytic function is discussed. We finally discuss the rationale to consider phagocytic processes as a therapeutic target to prevent or slow down dopaminergic degeneration.

## Introduction

Microglia are brain professional phagocytes mainly finalized to clearance of apoptotic or necrotic cells (Green et al., 2016) and removal of unfolded proteins such as amyloid beta (Aβ) or neuromelanin. Moreover, microglia participate in remodeling of neuronal connectivity by engulfment of synapses, axonal and myelin debris (Paolicelli et al., 2011) and combat central infections by direct phagocytosis of bacteria and viruses (Nau et al., 2014). These functions are carried by both unchallenged microglia in the developing brain and reactive microglia in pathological conditions (Sierra et al., 2010, 2013). Phagocytosis is part of the innate immune response of microglia, but also it mediates the adaptive responses by contributing to antigen presentation (Litman et al., 2005).

Phagocytosis is traditionally regarded as beneficial for tissue homeostasis by rapidly clearing dying cells, preventing the spillover of proinflammatory and neurotoxic molecules (Green et al., 2016; Wolf et al., 2017). In this context, an increased phagocytic activity was correlated with enhanced production of anti-inflammatory and decreased production of pro-inflammatory cytokines in microglia (Fadok et al., 1998; Wolf et al., 2017). However, the current view is that different targets and related receptors finely tune microglia responses, which appear as a continuum of multiple activation states (Hanisch and Kettenmann, 2007; Sierra et al., 2013; Wolf et al., 2017). For instance, phagocytosis of apoptotic neurons mediated by microglial triggering receptor expressed on myeloid cells-2 (TREM-2) was associated with decreased production of pro-inflammatory cytokines (Takahashi et al., 2005), while myelin debris phagocytosis enhanced the pro-inflammatory and dampened the anti-inflammatory profile in microglia (Siddiqui et al., 2016).

Microglia phagocytosis is still poorly explored in terms of functional consequences and intracellular machinery involved, but recent findings indicate that phagocytosis is defective in Alzheimer’s disease (AD; Lucin et al., 2013; Han et al., 2017; Krasemann et al., 2017) and might be dysregulated in other neurodegenerative disorders by genetic defects. Accordingly, p.R47H variant of TREM-2 is associated with Parkinson’s disease (PD; Rayaprolu et al., 2013).

This mini-review will focus on current understanding of the role of phagocytosis in PD, and how it is regulated at the physiological and molecular level and it will discuss whether phagocytotic activity might be considered a target for therapeutic intervention in PD.

## Molecular Mechanisms of Phagocytosis

The most important functional similarity between microglia and macrophages is their ability to perform phagocytosis, involving the three main steps “find-me”, “eat-me” and “digest-me” (Sierra et al., 2013; Wolf et al., 2017). The process is initiated by the activation of several membrane receptors, which directly recognize the target to engulf. Target-recognizing receptors show a certain degree of specificity toward signaling molecules exposed on the surface of their targets (pathogens, dead cells or protein aggregates) such as phosphatidylserine, oligosaccharides or heat-shock proteins (HSPs). Accordingly, the toll-like receptors (TLRs) in complex with scavenger receptors such as CD14, have been related to pathogen recognition, but are also involved in α-synuclein (α-SYN) uptake (Stefanova et al., 2011; Venezia et al., 2017). TAM (Tyro3, Axl and Mer) receptors recognize mainly apoptotic cells and virus-infected cells exposing phosphatidylserine (Fourgeaud et al., 2016; Tufail et al., 2017). TREM-2 signals the internalization of both dead cells and protein aggregates such as Aβ (Cho et al., 2014; Han et al., 2017; Krasemann et al., 2017). In addition, many other known and unknown receptors participate in target internalization and help to elaborate both effector and immunomodulatory responses (Litman et al., 2005). Different receptors trigger different signaling pathways that stimulate F-actin polymerization and phagosome formation (Arcuri et al., 2017).

The mechanistic features of macrophage phagocytosis have been extensively studied in past years (Green et al., 2016), but the molecular machinery that coordinate engulfment and digestion of dead cells and protein aggregates by microglia, relevant for neurodegenerative diseases, only recently have become an area of growing interest (Plaza-Zabala et al., 2017). Due to poor understanding of molecular mechanisms of microglial phagocytosis, it is assumed that they are similar, if not identical among phagocytes of myeloid linage (Plaza-Zabala et al., 2017). Based on how the phagosomes are formed, we can distinguish three main types of phagocytosis: LC3 (microtubule-associated protein 1A/1B-light chain 3)-dependent (LAP), LC3-independent phagocytosis and xenophagy, a specialized type of autophagy.

**Figure 1:**
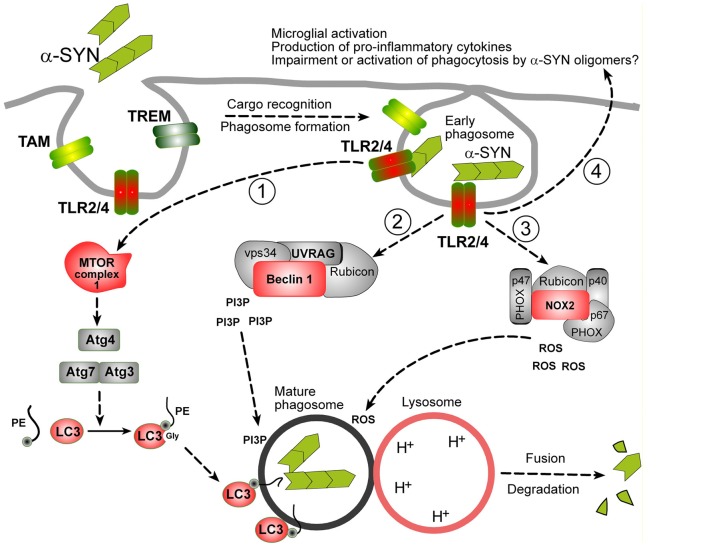
Possible mechanisms of α-synuclein (α-SYN) oligomers phagocytosis in microglia. It is assumed that microglial phagocytosis is run by the same molecular machinery as in macrophages, partially overlapping with autophagy machinery. Proteins in red: documented evidence for an involvement in microglial phagocytosis, proteins in gray: possible role, not yet documented. Phagocytosis is initiated by the recognition of a cargo by specific phagocytosis receptors (TLRs, TREMs, TAMs or others). In case of α-SYN oligomers TLR2 and TLR4 are engaged. These receptors trigger at least three distinct molecular pathways leading to the production of: (1) lipidated-LC3 family proteins; (2) phospholipids (Phosphatidylinositol 3-phosphate, PI3P); and (3) second messengers (ROS), necessary for the delivery and fusion of phagosomes with lysosomes and degradation of the cargo. Lipidated-LC3 family proteins are produced by a cascade of events starting from unknown upstream events (likely mammalian target of rapamycin complex 1 (mTORC1) inhibition, not shown), leading to the activation of Atg3/Atg7 complex and Atg4 involved in the cleavage and lipidation of LC3 family precursors. BECN-1/Beclin-1 complex, in association with Rubicon is involved in PI3P production. ROS are produced by the activation of NOX complex, composed of PHOX subunits (p40, p47 and p67) and NOX2 and Rubicon. Beside activation of phagocytosis, TLR2/4 receptors lead to the activation to other biological responses, like indicated at the end of arrow 4. See text for details.

LAP is triggered when a pathogen or dead cell, engaged by target recognizing receptors during phagocytosis, induces the translocation of autophagy machinery to the cargo-containing single-membrane phagosome (Martinez et al., 2015; Green et al., 2016). Three major signaling complexes are activated during LAP (see Figure 1). The aim of the first pathway is to ensure the production of lipidated-LC3 family proteins, which can embed in phagosomes, allowing their fusion with lysosomes (Martinez et al., 2015). The second pathway is Beclin-1 (BECN1) complex operating in association with Rubicon, Vps34 (Phosphatidylinositol 3-*kinase* class III), UV resistance-associated gene (UVRAG) and other enzymes, which are involved in the production of phosphatidylinositol 3-phosphate (PI3P), required for phagosome maturation (Wong et al., 2017). The third well-described protein complex activated by target-recognizing receptors is NADPH-oxidase type 2 (NOX2) module ensuring the superoxide production, required both for the cargo digestion and for stimulation of phagocytosis/autophagy machinery (Dodson et al., 2013; Martinez et al., 2015).

So far, LAP has not been characterized in microglia as such, but increasing evidence suggest that it may play a role in microglial phagocytosis. First, LC3 and autophagy have been recently implicated in α-SYN uptake and degradation, together with DJ-1, which is a product of *PARK7* gene and an autophagy regulator (Janda et al., 2012; Nash et al., 2017). Processing and lipidation of LC3 into LC3-II is mediated by ATGs (AuTophagy-related Gene products) proteins, which is negatively regulated presumably by mammalian target of rapamycin complex 1 (mTORC1) in microglia. Recently, mTORC1 has been shown to play a role in the regulation of autophagy (and possibly phagocytosis) mediated by TREM-2 (Ulland et al., 2017). Second, BECN1 was shown to be required for efficient microglial phagocytosis *in vitro* and in mouse brains and to be downregulated in brains of AD patients (Lucin et al., 2013). Finally, NOX2 is expressed in microglia and plays an established role in phagocytosis (Roepstorff et al., 2008; Rocha et al., 2016).

The alternative mechanisms of phagocytosis, either independent of LC3 and ATGs 5 and 7 (Cemma et al., 2016) or totally autophagy-dependent (xenophagy; Plaza-Zabala et al., 2017), have been described so far only in macrophages.

## Microglia: Emerging Roles in PD

After the first report of persistent microgliosis in post-mortem PD brain, a large amount of literature was produced in the attempt to elucidate the phenotype acquired by chronically reactive microglia (Gerhard, 2016; Joers et al., 2017). Microglia in PD maintain an uncontrolled pro-inflammatory phenotype, responsible for the progression of neurodegeneration. Pro-inflammatory cytokines together with iNOS induction, reactive oxygen species (ROS) and reactive nitrogen species (RNS) production, have been found in brain, cerebrospinal fluid and blood of PD patients and in experimental PD (Sawada et al., 2006; Mogi et al., 2007; López González et al., 2016; Joers et al., 2017). In addition, microglia in PD brains and rat *Substantia Nigra* (SN) overexpressing α-SYN showed a significant increase of IgG immunostaining (He et al., 2002; Orr et al., 2005; Theodore et al., 2008).

Furthermore, several studies have suggested that microglia may dynamically change phenotype in PD depending on disease-stage, which may account for the coexistence of pro- and anti-inflammatory molecules described in PD (Sawada et al., 2006; Mogi et al., 2007; Pisanu et al., 2014; Joers et al., 2017). Moreover, gene expression of cytokines and mediators of the immune response are region and stage-dependent in PD (López González et al., 2016). In the 1-methyl-4-phenyl-1,2,3,6-tetrahydropyridine (MPTP)-induced progressive model of PD, pro-inflammatory microglia gradually increase and prevail over anti-inflammatory microglia in presence of massive dopaminergic degeneration (Pisanu et al., 2014). Therefore, in the early PD stage both pro- and anti-inflammatory microglia may coexist, while in late stages they lose their capability to assume repair functions and unremitting pro-inflammatory microglia prevail.

Finally, the interaction of α-SYN with microglia represent a key event that leads to the unremitting shift of microglia to pro-inflammatory phenotypes (Austin et al., 2006; Theodore et al., 2008; Roodveldt et al., 2010; Ingelsson, 2016). α-SYN is prevalently expressed physiologically as a monomeric form, while in PD it aggregates in oligomers which are converted into mature amyloid fibrils, main components of Lewy bodies and neuritis (Ingelsson, 2016). α-SYN aggregates are also present in the extracellular biological fluids in PD patients (Spillantini et al., 1997; Tokuda et al., 2010; Majbour et al., 2016; Vivacqua et al., 2016; Visanji et al., 2017). Importantly, exposure to human α-SYN directly activates microglia, and the PD-relevant mutations of α-SYN A30P, E46K and A53T are more potent than wild type (Wt) α-SYN in triggering inflammatory responses (Klegeris et al., 2008). Transgenic mice overexpressing human A53T α-SYN develop chronic neuroinflammation and progressive degeneration together with microglia-derived oxidative stress (Gao et al., 2011). Thus, extracellular α-SYN is clearly involved in microglia activation and it has a profound impact on phagocytosis as discussed below.

A determining role in shaping microglia in PD is played by TREM-2, selectively expressed by microglia and involved in modulating inflammatory responses and in the phagocytosis of apoptotic neurons (Jay et al., 2017). Stimulation or overexpression of TREM-2 increases, while knockdown inhibits phagocytosis of apoptotic neurons and increases pro-inflammatory gene transcription (Takahashi et al., 2005). Overexpression of TREM-2 is neuroprotective and reduces inflammation in MPTP-intoxicated mice through inhibition of the TLR4-mediated activation of nuclear factor (NF)-κB signaling (Ren et al., 2018). The fractalkine receptor CX3CR1, specifically expressed in microglia, is also involved in PD neuropathology. Through binding to neuronal CX3CL, CX3CR1 plays a fundamental role in the microglia-neurons communication (Harrison et al., 1998), being involved in homeostatic maintenance of microglia in the quiescent state, regulation of chemo-attraction and synaptic pruning/maturation (Paolicelli et al., 2011; Mecca et al., 2018). Therefore, CXCL-CX3CR1 profoundly affects microglial-mediated inflammatory responses and neurotoxicity (Sheridan and Murphy, 2013). In PD models, MPTP-intoxicated mice with CX3CR1 deficiency displayed aggravated pathology and greater loss of tyrosine hydroxilase-immunoreactive (THIR) neurons in the SNc (Cardona et al., 2006). Moreover, in the intrastriatal 6-hydroxydopamine (6-OHDA) rat model of PD, the continuous delivery of recombinant CX3CL1 suppressed microglia activation and reduced neuronal loss (Pabon et al., 2011).

## Microglia Phagocytic Function in PD

Several studies described an altered phagocytic function of microglia in PD (Table 1). Pro-inflammatory and phagocytic microglia with increased Major Histocompatibily Complex (MHC) II expression was described in MPTP-treated monkeys and mice (Barcia et al., 2011, 2013; Depboylu et al., 2012). Upon MPTP administration, mouse microglia polarize to contact and phagocytose damaged dopaminergic neurons (Barcia et al., 2012). The increased engulfment and phagocytosis were suggested to contribute to degenerative processes (Barcia et al., 2013). Accordingly, blocking phagocytosis preserved live neurons from inflammation-induced cell death (Fricker et al., 2012). In contrast, we recently found that MPTP-induced neurodegeneration in mice was associated with decreased expression of scavenger receptor Mannose Receptor C-Type 1 (MRC1), while peroxisome proliferator activated receptor gamma (PPARγ)-mediated neuroprotection was associated with increased MRC1 expression and phagocytosis, suggesting a beneficial role of phagocytosis (Lecca et al., 2018).

**Table 1:** Current evidence of altered phagocytosis in PD and experimental PD models.

Model	Alterations of phagocytosis	Reference
**Human studies**		
Post-mortem brain tissue	Increased expression of microglial CD68 in SN	Croisier et al. (2005) and Doorn et al. (2014)
Peripheral immune cells	Defective phagocytosis of beads in monocytes and fibroblasts	Salman et al. (1999) and Gardai et al. (2013)
**Animal models**		
Microglia from α-SYN knock-out mice	Increased expression of CD68, impaired phagocytic function	Austin et al. (2006)
MPTP-treated monkeys and mice	Microglia with phagocytic features in SN	Barcia et al. (2011, 2013) and Depboylu et al. (2012)
A53T α-SYN overexpressing mice	Increased expression of Ax1 TAM in spinal cord microglia	Fourgeaud et al. (2016)
MPTP-treated mice	Increased expression of MRC1	Lecca et al. (2018)
BV-2 cells, rat primary microglia	Monomeric α-SYN increases phagocytosis of microspheres Oligomeric α-SYN decreases phagocytosis of microspheres	Park et al. (2008)
Primary microglia	WT and A53T α-SYN increase phagocytosis of microspheres A30P and E46K α-SYN decrease phagocytosis of microspheres	Roodveldt et al. (2010)
BV-2 cells	A53T α-SYN decreases phagocytosis of bioparticles	Rojanathammanee et al. (2011)
Primary microglia	Soluble or fibrillar α-SYN increases phagocytosis of microspheres	Fellner et al. (2013)
Primary microglia	Adult microglia phagocytoses oligomeric α-SYN less efficiently than young microglia	Bliederhaeuser et al. (2016)
Primary microglia	Rotenone increases phagocytosis of microspheres	Emmrich et al. (2013)
LPS-treated MMGT12 cells	PPAR-γ agonist increases the expression of CD68 and the phagocytosis of beads or 6-OHDA-necrotic SH-SY5Y cells	Lecca et al. (2018)

Few studies focused on the immunohistochemical evaluation of CD68, a macrophagic protein and suggested marker of phagocytosis. Increased CD68 expression was described in the parkinsonian SN (Croisier et al., 2005; Doorn et al., 2014), and confirmed in the α-SYN overexpressing rat model (Table 1). In one study CD68 increased early prior to neurodegeneration (Theodore et al., 2008), while in another study correlated with dopamine neurons death (Sanchez-Guajardo et al., 2010). The upregulation of Axl TAM phagocytic receptor was reported in the spinal cord microglia of A53T α-SYN mouse, and loss of this receptor slightly extended survival (Fourgeaud et al., 2016), suggesting that microglia phagocytosis of motor neurons may hasten death of mice.

Different conclusions were reached by studies addressing microglial phagocytic function via functional assays, such as phagocytosis of beads or apoptotic cells (Table 1). Microglial phagocytosis but not inflammation was induced by rotenone and rotenone-induced neurotoxicity was prevented by phagocytosis inhibitors (Emmrich et al., 2013). Similarly, anti-inflammatory drug ibuprofen inhibited phagocytosis of dead neurons and NO production by microglia (Scheiblich and Bicker, 2017). However, a significant defect in phagocytic function was observed in fibroblasts and in monocytes of PD patients (Salman et al., 1999; Gardai et al., 2013).

Since phagocytosis has been traditionally regarded as a beneficial event associated with the anti-inflammatory phenotype of microglia, this evidence queries how relevant this assumption is in neurodegenerative diseases, where microglia display an abnormal inflammatory profile.

Studies dissecting the interaction of different α-SYN forms with microglia strongly implicate α-SYN in altered phagocytosis. These studies have highlighted the role of α-SYN variants on the induction of microglial phenotypes with abnormal phagocytic function. Microglia incubated with A53T α-SYN displayed a pro-inflammatory profile and impaired phagocytosis (Rojanathammanee et al., 2011). In contrast, Roodveldt et al. (2010) showed that both Wt and A53T α-SYN promoted phagocytosis in microglial cells, while the A30P and E46K α-SYN induced opposite effect. Interestingly, Wt α-SYN was also associated with moderate inflammatory response, indicating the coexistence of pro-inflammatory and phagocytic profiles, and suggesting that a combination of alternative and classical activation states may occur in microglia (Roodveldt et al., 2010). However, microglia from α-SYN knock-out mice displayed increased basal and LPS-stimulated production of pro-inflammatory cytokines and expression of CD68, but impaired phagocytosis, suggesting that physiological levels of α-SYN prevent inflammation and promote phagocytosis (Austin et al., 2006). α-SYN conformation impacts microglial phagocytosis, with monomeric α-SYN stimulating, while oligomeric α-SYN inhibiting both basal and LPS-stimulated phagocytosis (Park et al., 2008). In addition, microglia phagocytosis was augmented, together with production of ROS and pro-inflammatory cytokines after treatment with soluble or fibrillar α-SYN, confirming the occurrence of mixed phenotypes in pathological conditions (Fellner et al., 2013). Finally, nitrated α-SYN increased both pro-inflammatory cytokines and the anti-inflammatory cytokine IL-10 in primary microglia (Reynolds et al., 2009). Importantly, age is a crucial factor for microglial phagocytosis, since microglia from adult mice was less efficient to engulf oligomeric α-SYN than young mice, while responding with higher TNFα release (Bliederhaeuser et al., 2016). Therefore, studies indicate a functional specificity for α-SYN conformational variants. In this regard it is important to note that in extracellular fluids of PD patients, the coexistence of multiple α-SYN conformations has been reported, with prevalence of pathological oligomeric α-SYN (Tokuda et al., 2010; Majbour et al., 2016).

The role of TLRs as mediators of α-SYN-effects on microglia is emerging, indicating a role in both inflammatory responses and phagocytosis. Aggregated but not fibrillar or monomeric α-SYN directly activated microglia through TLR2, leading to production of inflammatory mediators (Kim et al., 2013). The TLR4 was also suggested to mediate microglia phagocytosis of β-SYN (Stefanova et al., 2011). Both the TLR2 and TLR4 were elevated in peripheral immune cells and in PD brain, where TLR2 colocalized with microglia (Doorn et al., 2014; Drouin-Ouellet et al., 2014). The coexistence of α-SYN conformations and TLRs stimulation may lead to a microglia phenotype with inflammatory and phagocytic functions, which may be harmful for neurons.

A role of TREM-2 in promoting phagocytosis has been well characterized for *in vitro* clearance of Aβ and *in vivo* models of AD (Taylor et al., 2017). In contrast, the role of TREM-2 in PD-associated dysfunctional phagocytosis has not been investigated. Defective function of TREM-2 in PD may lead to incomplete removal of apoptotic cells and debris and accumulation of toxic products that may chronically stimulate microglia to release cytotoxic species. Whether TREM-2 is affected by α-SYN accumulation is unknown.

Recent studies suggested that the CXCL-CX3CR1 axis modulates the inflammatory response induced by α-SYN overexpression. CX3CR1−/− mice displayed a reduced α-SYN-mediated inflammatory response, with reduced microglia phagocytosis (Thome et al., 2015).

Therefore, while studies point to a dysregulation (either up- or downregulation) of phagocytosis in microglia as a prominent event in the PD neuropathology, quantitative and qualitative α-SYN abnormalities emerge as the underpinning mechanisms.

## Current Gaps and Future Perspectives

While it is clear that the shift to pro-inflammatory phenotypes contributes to neurodegeneration, there is no consensus on the role of phagocytosis in PD and research in this field presents several gaps.

An important gap is related to its molecular mechanisms. Since microglia and macrophages share several functional and surface-receptor similarities, it has been assumed that the mechanistic features of phagocytosis should be the same in both cell types (Plaza-Zabala et al., 2017). However, beside some progress in our understanding of phagocytosis machinery involved in Aβ clearance (Krasemann et al., 2017; Sarlus and Heneka, 2017), it is unknown what types of phagocytosis can be activated in microglia, whether other microglia-specific types of phagocytosis exist, and what is the role of autophagy in this process. All these issues are heavily investigated in macrophages, but not yet in microglia, and future efforts will clarify which are the common mechanisms and targetable differences.

Most important, it is still unclear whether phagocytosis is pathologically activated or rather defective in PD. The answer to this question might be hampered by our incomplete understanding of microglial plasticity and its regulation, especially in the context of progressing PD. The current literature strongly suggests that microglia acquire mixed phenotypes in PD displaying an altered phagocytic activity, which escape from traditional classification in pro- and anti-inflammatory phenotypes. Recent innovative studies support a beneficial effect of phagocytosis stimulation in PD (Venezia et al., 2017; Lecca et al., 2018). Additional studies are needed to understand whether we can pharmacologically restore phagocytosis homeostatic levels. Considering that a prompt clearance of dead cellular bodies and protein aggregates should be beneficial in PD, we expect that this concept may prevail.

The current debate on phagocytosis in PD resembles a long-lasting debate about a role of autophagy in this pathology. Despite genetic evidence pointed toward a positive function of autophagy, experimental evidence often indicated a hyperactivation of autophagy in PD (Banerjee et al., 2010; Janda et al., 2012; Dagda et al., 2013). We understood now that conflicting results were often caused by technical limitations and misleading interpretation (Janda et al., 2012), and current view favors a beneficial role of autophagy in PD, while its pharmacological stimulation has become an achievable goal (Janda et al., 2015; Moors et al., 2017). Considering many functional and mechanistic similarities between autophagy and phagocytosis, it is safe to speculate that concomitant stimulation of both pathways in different cellular compartments, will became a therapeutic target in the future.

## Author Contributions

ARC addressed current knowledge of phagocytosis in PD and in PD models. EJ addressed all the molecular aspect of phagocytosis, both in general and in relation to PD. LB reviewed the literature on alpha-syn and phagocytosis and addressed this specific issue in the review.

## Conflict of Interest Statement

The authors declare that the research was conducted in the absence of any commercial or financial relationships that could be construed as a potential conflict of interest.

## Abbreviations

α-SYN: α-synuclein
6-OHDA: 6-hydroxydopamine
Aβ: amyloid beta
AD: Alzheimer disease
CNS: central nervous system
CPu: *Caudate putamen*
CX3CL: fracktaline receptor ligand
CX3CR1: fracktaline receptor
HSP60: Heat shock protein 60
MHC: Major Histocompatibily Complex
MPTP: 1-methyl-4-phenyl-1,2,3,6-tetrahydropyridine
PD: Parkinson disease
PI3P: phosphatidylinositol 3-phosphate
PPARγ: peroxisome proliferator activated receptor gamma
RNS: reactive nitrogen species
ROS: reactive oxygen species
SN: *Substantia Nigra*
TH-IR: tyrosine hydroxilase-immunoreactive
TREM-2: microglial triggering receptor expressed on myeloid cells-2
Wt: wild type.
